# Fang-Ji-Huang-Qi-Tang Attenuates Degeneration of Early-Stage KOA Mice Related to Promoting Joint Lymphatic Drainage Function

**DOI:** 10.1155/2020/3471681

**Published:** 2020-03-20

**Authors:** Haihui Han, Yinghui Ma, Xiaoyun Wang, Ruisheng Yun, Sheng Lu, Mengxiong Xia, Yongjun Wang, Qi Shi, Weitao Zhai, Qianqian Liang, Hao Xu

**Affiliations:** ^1^Longhua Hospital, Shanghai University of Traditional Chinese Medicine, Shanghai, China; ^2^Guanghua Hospital, Shanghai University of Traditional Chinese Medicine, Shanghai, China; ^3^Key Laboratory of Theory and Therapy of Muscles and Bones, Ministry of Education, Shanghai, China

## Abstract

Osteoarthritis (OA) is a chronic degenerative joint disease characterized by the breakdown of articular cartilage, subchondral bone remodeling, and inflammation of the synovium. In this study, we investigated whether Fang-Ji-Huang-Qi-Tang (FJHQT) decoction improved the joint structure of OA or delayed the process of knee joint degeneration in OA mice by promoting lymphatic drain function. The mice were randomly divided into four groups, the sham group, the PBS group, the FJHQT-treated group, and the Mobic-treated group. The mice in each group were tested for lymphatic draining function at 4, 6, 8, and 10 weeks postsurgery (WPS), then sacrificed (*N* = 10/group). Using a near-infrared indocyanine green (NIR-ICG) lymphatic imaging system, we found that the lymphatic drain function was significantly reduced in the PBS group compared with the sham group. After treatment with the FJHQT decoction, the lymphatic draining function improved at 4 wps and 6 wps. The results of the analysis indicated a strong correlation between lymphatic draining function (ICG clearance) and the degree of joint structural damage (OARSI score). By Alcian blue/orange G (ABOG) staining of the paraffin sections, the FJHQT-treated group exhibited less cartilage destruction and lower OARSI scores. Moreover, the result of immunohistochemical staining (IHC) shows that FJHQT decoction increased the content of type II collagen in knee joints of OA mice at 4 wps and 6 wps. By the double immunofluorescence staining of podoplanin and smooth muscle actin in the paraffin sections, the capillaries and mature lymphatics in the FJHQT group increased at 4 wps. In conclusion, the FJHQT decoction can increase lymphatic vessel number, promote joint lymphatic draining function, and postpone knee osteoarthritis pathologic progression in the early stage of a collagen-induced mouse model. Therefore, the application of sufficient lymphatic drainage in the knee joint may be a new treatment method for knee joint osteoarthritis (KOA).

## 1. Introduction

Osteoarthritis (OA), characterized by a progressive loss of cartilage and subchondral bone, is a degenerative joint disease leading to disability in old people [1]. The treatment of OA is still dominated by nonsteroidal anti-inflammatories; however, the long-term use of nonsteroidal anti-inflammatories usually causes severe gastrointestinal side effects, especially in the elderly [2, 3]. Moreover, in clinical practice, there are still some difficulties in controlling the clinical symptoms of osteoarthritis, even when the best combination of drugs and nondrug interventions is given [4]. Therefore, searching for effective and safe treatment options for OA is still very urgent in clinical work.

Previous studies have shown that the function of lymphatic drainage plays an important role in the occurrence and development of osteoarthritis. The lymphatic system maintains fluid balance and participates in lipid transport under normal physiological conditions. In addition, it is closely related to tumor metastasis, wound healing, and inflammatory response [5]. Zhou et al. [6] found that a large number of new lymphatic vessels appeared in the chronic stage of arthritis. Zhang et al. [7] found inflammation stimulated lymphangitic hyperplasia in periarticular tissues and locally transported lymph nodes transport. Shi et al. [8] found that the number of lymphatic capillaries around the knee joints in KOA model mice increased at 12 weeks after modeling and decreased significantly at 20 weeks. These results have proven that there were abnormal changes in the structure and function of the lymphatic vascular system in the OA joint. Lymphatic vessel formation is a compensatory response to joint inflammation, which may drain inflammatory cytokines away from the OA joint, such as TNF-alpha that could increase MMP-13 gene expression by activating ERK, p38, in addition to MAP kinases, and AP-1 and NF-kappa B transcription factors. Therefore, the lymphatic vascular system plays a critical role in the OA pathologic process. For thousands of years, traditional Chinese medicine has been used to treat Chinese patients, including articular disease cases. The FJHQT decoction, invented by the ancient Chinese medical doctor Zhongjing Zhang, is a classic formula that has the function of clearing the dampness and congestion in the joint.

In this study, the effectiveness of FJHQT decoction on knee joint osteoarthritis (KOA) was evaluated by treating collagen-induced KOA model mice, detecting the collagen type II expression of joint cartilage, and analyzing the changes in the lymphatic draining function of the knee joint. In addition, we analyzed the correlation between lymphatic draining function and joint cartilage degeneration, which may illustrate how it is possible to strengthen joint lymphatic draining functions. This method could be a promising way to prevent or postpone OA for disease modification.

## 2. Methods

### 2.1. Animals and Models

Ten-week-old male C57BL/6J mice were obtained from the Shanghai Model Organisms Center (Shanghai, China) and maintained under conventional housing conditions. All murine studies were performed according to the protocols approved by the Institutional Animal Care and Use Committee (IACUC) at the Shanghai Model Organisms Center and followed the guidelines of the Animal Welfare Act, license number SCXK (Shanghai) 2018-0026. To induce OA, 1 U of highly purified bacterial type VII collagenase (Sigma-Aldrich) in 6 *μ*l of physiological saline (vehicle) was injected into the right knee joint of mice twice over 2 consecutive days (totaling 2 U) [9, 10]. Animals were anesthetized using isoflurane and given a subcutaneous injection of meloxicam (2 mg/kg body weight) prior to collagenase injection. After modeling, mice (*n* = 10/group) were randomly separated into three groups: the FJHQT group, the Mobic group, and the PBS group. The left knee joints of mice were used as a sham group. All mice were treated with different drugs by oral gavage every other day.

### 2.2. Plant Material and Drug Preparation

Herbs in the FJHQT decoction included Fangji (Radix Stephaniae Tetrandrae) 12 g, Huangqi (Radix Astragali Mongolici) 15 g, Baizhu (Rhizoma Atractylodis Macrocephalae) 9 g, and Gancao (Radix Glycyrrhizae) 6 g and were authenticated by a pharmacognosist according to standard protocols and prepared by Longhua Hospital affiliated to Shanghai University of Traditional Chinese Medicine. The mouse (20 g) and human (60 kg) were converted to conventional Chinese medicine dosage (experimental animal pharmacology: conversion coefficient 9.01), and the mice were treated with a concentration of 0.63 g/mL. Five doses of the above traditional Chinese medicine were soaked in 12 times the volume of water (2520 mL) for 30 min and boiled for 30 min; the drug solution was filtered, the filter residue was boiled in 8 times the volume of water (1680 mL) for another 30 min, and the solution was filtered again. The mixture from the two filtrates was heated and condensed to a volume of 335 mL under reduced pressure to obtain the concentration of FJHQT decoction and then stored at −20°C until use. Mice were given 0.2 ml FJHQT decoction with a concentration of 0.63 g on alternate days. In this study, we used Mobic (Boehringer Ingelheim, German), a common nonsteroidal anti-inflammatory drug for KOA, as a positive control drug in our experiments. The mice were treated with 0.2 ml of Mobic mixture with PBS at a concentration of 0.0225 mg/ml every other day.

### 2.3. Near-Infrared Indocyanine Green (NIR-ICG) Lymphatic Imaging

**Figure 1:**
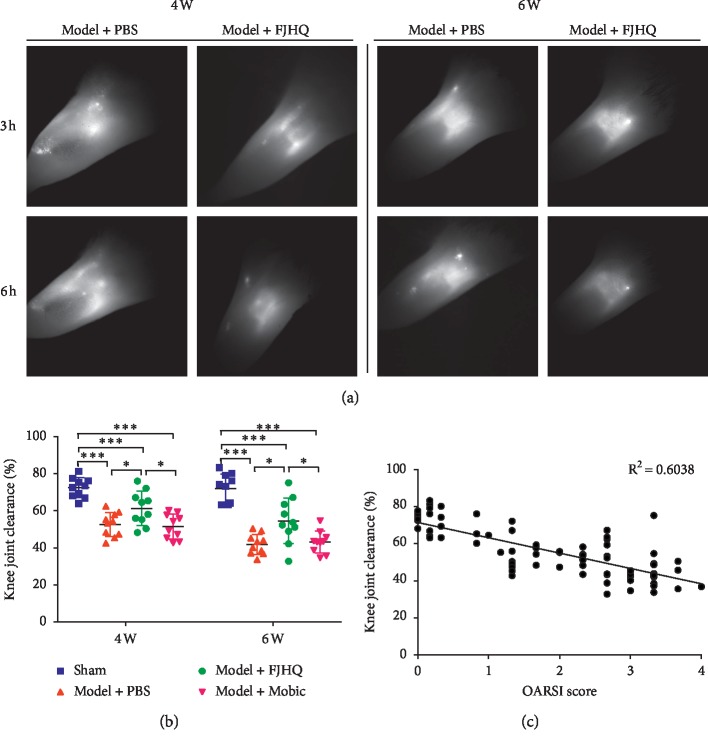
Near-infrared indocyanine green (NIR-ICG) lymphatic imaging at 3 hours and 6 hours (a). At 4 and 6 weeks, the color intensity of ICG in PBS group did not significantly change at 3 hours and 6 hours (b). After the medication, the color intensity of FJHQ decoction group decreased significantly in 3 hours and 6 hours (b). Correlation between lymph drainage (ICG clearance) and tissue damage (OARSI score) in knee joints (*R*^2^ = 0.6038 < 1). ^*∗*^*P* < 0.05,  ^*∗∗*^*P* < 0.01,  and ^*∗∗∗*^*P* < 0.001.

NIR-ICG lymphatic imaging was performed by using the Fluobeam 800 imaging system (Fluoptics, France) as described previously [6, 8, 11]. In short, the fur around the knee joints was removed with a depilatory cream. Using a microsyringe (Hamilton Inc., USA) and 30 G needle (BD Inc., USA), a 6 *μ*l solution of indocyanine green (Acorn) in distilled water (0.1 mg/ml) was injected into knee joints of mice. Then, the knee joints were photographed under an infrared laser after 3 and 6 hours. As shown in Figure 1, ICG fluorescence can be visualized in the knee joints under the NIR imaging system. Using ImageJ software (National Institutes of Health, Bethesda, MD, USA), regions of interest (ROIs) in the knee joints were identified and analyzed. Then, the data were put into the formula, and the result was the clearance rate of the mouse's knee joint, which could represent the lymphatic drainage function of the knee. The knee joint of clearance was calculated using the following equation: clearance=((ROI_3*h*_ − ROI_3hbg_) − (ROI_6 h_ − ROI_6 hbg_))/(ROI_3 h_ − ROI_3 hbg_)*∗*100%. where ROI_3h_ is the regions of interest at 3 hours postinjection, ROI_3hbg_ is the regions of interest of the background at 3 hours postinjection, ROI_6h_ is the regions of interest at 6 hours postinjection, and ROI_6hbg_ is the regions of interest of the background at 6 hours postinjection.

### 2.4. Histological Scoring

After near-infrared imaging, the mice were sacrificed, and the dissected knee joints were fixed in a 4% paraformaldehyde solution at room temperature for 48 hours and decalcified in a 10% EDTA solution for 14 days. Knee joint samples (*n* = 10/group) were paraffin-embedded and consecutively sectioned at 4 *μ*m thickness. A total of 20 sections that represented the entire joint were collected and divided into 3 levels. Each level was 28 *μ*m from the previous level. One section from each of the 3 levels was stained using Alcian blue/orange G (ABOG) staining. Slides were scanned using the Olympus VS120-S5-E whole-slide imaging system (Olympus, Japan). All images were analyzed using the Olympus OlyVIA software (Olympus, Japan). The scoring was performed based upon the semiquantitative grading system designed by the Osteoarthritis Research Society International (OARSI) working group with damage ranging from 0 (no damage) to 6 (complete denudation of the cartilage) [12]. For each knee analyzed, slides representing the entire joint were blinded and scored by 2 independent observers. We outlined ROIs, which included distal femur, tibia, meniscus, articular cartilage, synovial, ligaments, and adjacent soft tissues. The total articular cartilage area of the ROI was determined automatically with Olympus VS-120 software and ranged from 2.98^*∗*^10^5^ *μ*m^2^ to 4.54^*∗*^10^5^ *μ*m^2^. The data are presented as the mean from 3 levels cut from each joint sample.

### 2.5. Immunohistochemical Staining for Type II Collagen

Sectioned knee joint samples (*n* = 10/group) were pretreated in xylene to clear away the paraffin. Then, H_2_O_2_ was added to the slices for 10 minutes to reduce the activity of endogenous peroxide. The sections were incubated with proteinase K (Leagene, IH0310) for 10 minutes at 37°C to repair antigens and then inhibited by 4% bovine serum albumin-phosphate-buffered saline (BSA; BioFroxx, German; CAT. No : 4240GR100) for 1 hour to expose collagen epitopes. Antibodies against collagen II (1 : 1000; ab34712, Abcam, UK) diluted in 0.4% BSA were incubated with the samples overnight at 4°C. After rinsing in PBS, the slides were incubated with an antirabbit HRP-conjugated secondary antibody. The color reactions were finally performed using a peroxidase substrate (Vector Laboratories, Burlingame, CA 94010) after treatment of the sections with a mixture of avidin and biotinylated horseradish peroxidase (Vector Laboratories). Mayer's hematoxylin was used to counterstain the sections. Negative controls were included to check the proper specificity and performance of the applied reagents by omitting the primary antibody.

### 2.6. Immunofluorescence Staining

One section from the knee joint slices was chosen for immunofluorescence staining [13]. Briefly, deparaffinized sections were subjected to antigen retrieval by proteinase K, blocked in 4% BSA for 1 hour, and then incubated with hamster monoclonal antimouse podoplanin (Abcam Inc., Cambridge, MA, USA; cat. #ab11936; clone: 8.1.1, 1 : 1000) at 4°C overnight, followed by Alexa Fluor 546-conjugated goat antihamster (Invitrogen-Molecular Probes, Eugene, OR, USA; cat. #A21111, 1 : 400) and fluorescein isothiocyanate- (FITC-) conjugated antimouse *α*-SMA (1 : 400 dilution; Sigma) for 1 hour. The sections were mounted with an aqueous mounting medium, Vector H-1000, as previously described [7, 8]. Double immunofluorescence-stained slides were scanned using an Olympus VS-120 whole-slide imaging system as described previously [14] to evaluate capillaries and mature lymphatic vessels. To examine the distribution of periarticular lymphatic vessels in an entire knee, Podoplanin^+^/*α*-SMA^−^vessels were defined as lymphatic capillaries, and podoplanin^+^/*α*-SMA^+^ vessels were defined as collecting lymphatic vessels according to published literature [15, 16]. Images were analyzed using Olympus VS-120 software. We outlined the ROIs of the knee joints, which included synovial capsules, ligaments, and adjacent soft tissues (but excluded synovial space, articular cartilage, meniscus, and bones). The total tissue area of the ROI was determined automatically with Olympus VS-120 software. The area of lymphatic vessels within the entire ROI was measured with the lymphatic area divided by the total tissue area.

### 2.7. Statistical Data Analysis

Statistically significant differences between the groups were analyzed using Two-Way ANOVA, followed by the Tukey–Kramer post hoc test. Results are expressed as the mean ± standard deviation of the mean (SD) and considered significant when the *P*-value was <0.05. Statistical analysis was performed using GraphPad Prism 7.00 software (GraphPad Software Inc., La Jolla, CA, USA). Correlation among multiple variables was analyzed and plotted by MATLAB 2016a (MathWorks) software.

## 3. Results

### 3.1. FJHQT Decoction Promoted Joint Lymphatic Drainage Function in KOA Mice

Using near-infrared indocyanine green (NIR-ICG) lymphatic imaging system, we found no significant change in the function of lymphatic drainage in the sham group at 4 weeks (72.485 ± 5.384%) and 6 weeks (72.005 ± 7.901%). ICG clearance of OA knee joint at 4 weeks (52.597 ± 6.487%) and 6 weeks (41.851 ± 5.509%) was significantly reduced compared with the sham group (Figure 1(a)) while lymphatic drainage function improved at 4 weeks (61.31 ± 9.20%) and 6 weeks (54.537 ± 12.257%) after treatment with FJHQT decoction (Figure 1(a)). The difference was statistically significant (*P* < 0.05, Figure 1(b)).

### 3.2. FJHQT Decoction Decreased OARSI Score, Increased the Content of Cartilage Type II Collagen in the Osteoarthritis Knee Joint, and Postponed the Progression of Joint Degeneration in Osteoarthritis of the Knee

**Figure 2:**
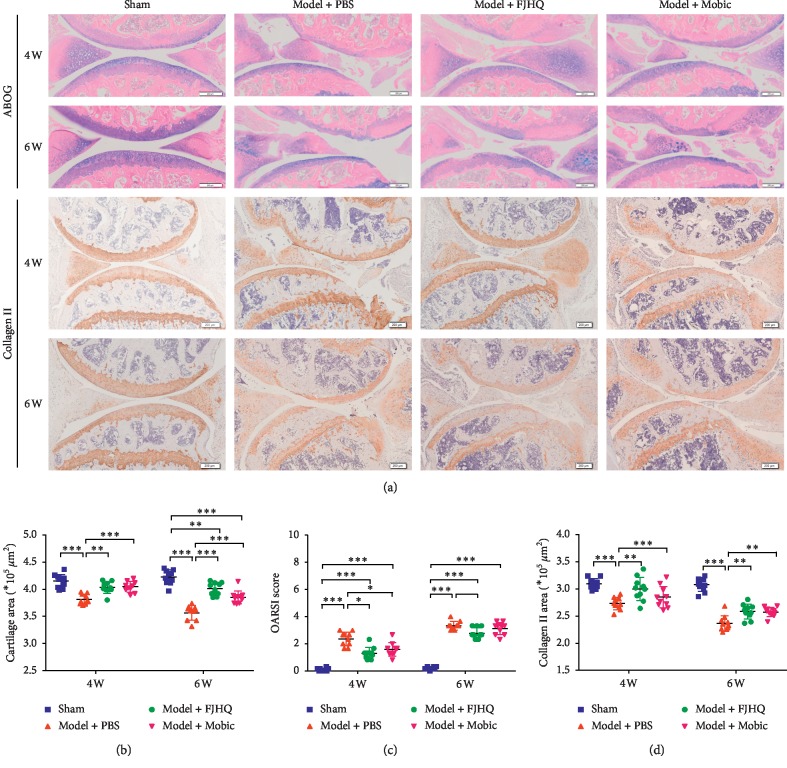
Alcian Blue Hematoxylin/Orange G staining (ABOG) (a) and IHC of type II collagen (a). Analysis of collagen II in articular cartilage (b) and tissue injury assessed by OARSI score (c). Analysis of articular cartilage area stained by histological sections of the knee in mice (d). Values are mean ± SD of 10 joints. Significance was determined by two-way ANOVA followed by Tukey's test (*P* < 0.05). ^*∗*^*P* < 0.05,  ^*∗∗*^*P* < 0.01, and ^*∗∗∗*^*P* < 0.001.

From the typical images of ABOG staining, we found that the joint structure of the sham group was basically unchanged over time (Figure 2(a)). However, with the change of time, the joint structure destruction of the model group gradually increased, and even the loss of half tibial plateau appeared at the end (Figure 2(a)). After FJHQT decoction treatment, the destruction of the knee joint structure in mice was less significant compared to the PBS group, and Mobic did not work as well as the FJHQT decoction. These findings were demonstrated by the OARSI scores for evaluating joint structure condition (Figure 2(c)). We analyzed the correlation between lymphatic drainage function (ICG clearance) and joint damage degree (OARSI score) and found a strong correlation between them (*R*^2^ = 0.6038, Figure 1(c)). By ABOG staining of the paraffin sections, we calculated the articular cartilage area and found that the articular cartilage area of the FJHQT group increased compared with the PBS group (4 weeks, 3.816 ± 0.092^*∗*^10^5^ *μ*m^2^; 6 weeks, 3.568 ± 0.134^*∗*^10^5^ *μ*m^2^). The difference was statistically significant (*P* < 0.05, Figure 2(a)). We investigated whether FJHQT decoction can delay the progression of joint degeneration by immunohistochemical staining of type II collagen (Figure 2(a)). Through the analysis of type II collagen content in articular cartilage, we found that the FJHQT group significantly increased at 4 and 6 weeks (Figure 2(d)).

### 3.3. FJHQT Decoction Can Increase the Number of Lymphatic Vessels around the Knee in the Early Stages of Osteoarthritis

**Figure 3:**
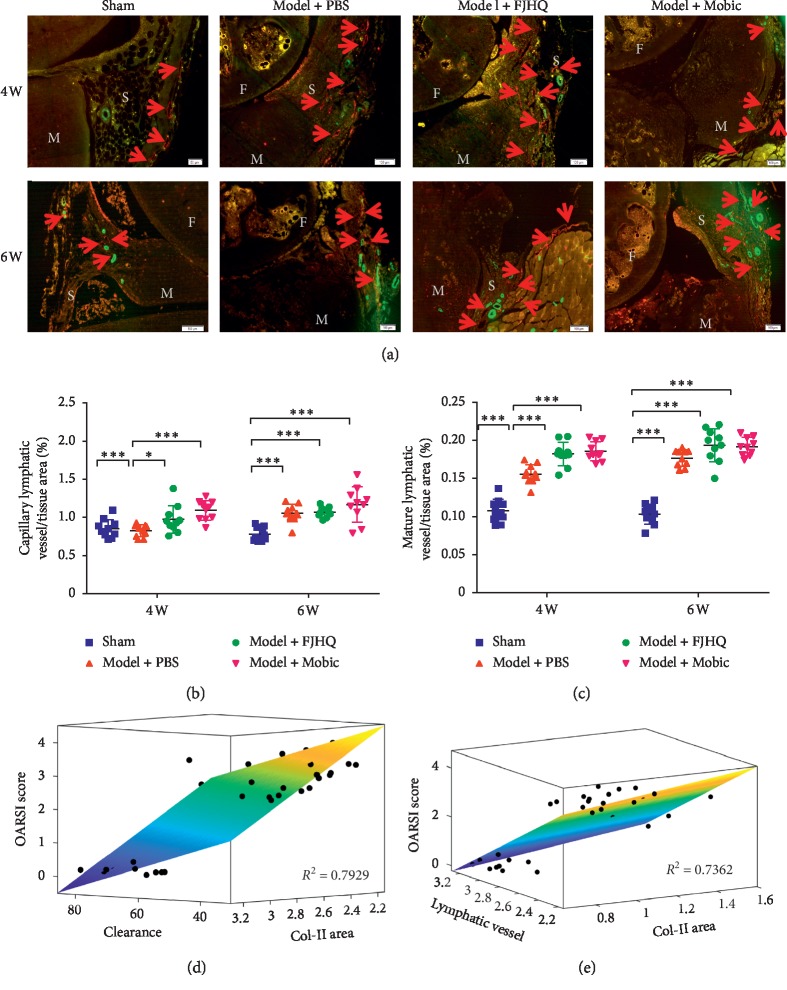
Immunofluorescence staining of the paraffin sections (a) and analysis of mature lymphatics (c) and capillary lymphatics (b). Correlation analysis of OARSI score with lymphatic drain function and type II collagen area (d). Podoplanin+/*α*-SMA− marks lymphatic capillaries and podoplanin+/*α*-SMA+ marks mature lymphatic vessels. Correlation analysis of OARSI score with the percentage of lymphatic capillaries and collagen type II area (e). Values are mean ± SD of 10 joints. Significance was determined by two-way ANOVA followed by Tukey's test (*P* < 0.05). ^*∗*^*P* < 0.05,  ^*∗∗*^*P* < 0.01,  and ^*∗∗∗*^*P* < 0.001.

To investigate the effect of FJHQT decoction on the generation of lymphatic vessels, we performed immunofluorescence double-staining on knee joint sections (Figure 3(a)). We performed a statistical analysis of the percentage of capillaries and mature lymphatic vessels in the periarticular tissue of the knee joint (Figure 3(b) and 3(c)). We found that the percentage of capillaries increased in the PBS group at 6 weeks and then decreased gradually in the OA knee joint. After FJHQT treatment, the percentage of capillaries increased in the fourth week compared with the PBS group (Figure 3(b)). Similarly, the mature area percentage in the FJHQT group also increased in the fourth week compared with the PBS group, and the difference was statistically significant (Figure 3(c)). We analyzed the correlation among the OARSI score and lymphatic drainage function and collagen type II area and found that there was a correlation between the 3 outcome measures (Figure 3(d)). In addition, there was a correlation among the OARSI score and the percentage of lymphatic capillaries and collagen type II area (Figure 3(e)).

## 4. Discussion

At present, nonsteroidal anti-inflammatory drugs (NSAIDs) are still the first choice for KOA treatment, and there is still no effective drug to control the disease completely [4]. However, it has become clear that OA is a disease of the entire joint rather than simply the cartilage alone. An increasing amount of evidence highlights the association between factors produced by the lymphatic system and the initiation and progression of the disease [7, 8, 17–19]. Through this investigation, we hope to find a drug that can postpone the process of osteoarthritis and provide more options for the treatment of OA. A mechanism has been proposed in our previous study to explain the onset of osteoarthritis of the knee [8, 20, 21]. FJHQT decoction, an ancient Chinese medical mainstay, has been the most popular prescription for treating edema and arthralgia of rheumatism in the Traditional Chinese Medical system for more than 1800 years [22, 23]. In our current study, we demonstrated that FJHQT decoction could delay the progression of osteoarthritis by regulating lymphatic drain function.

In this study, type VII collagenase was used to induce the mouse KOA model [10]. Compared with other modeling methods, the KOA model induced by type VII collagenase has the advantages of a high modeling success rate and short modeling time for joint trauma and other conditions. Literature reports have shown that mice can gradually develop KOA-like pathological changes 2 weeks after the first injection [9]. Four weeks after the induction of the KOA model, the characteristic changes of articular cartilage destruction, such as cartilage loss, calcification, and cartilage erosion, were observed in the PBS model group. We used the OARSI scoring system to evaluate the severity of structural damage of knee joints in the 4 experimental groups [12]. The injury extent ranged from 0 (no injury) to 6 (complete cartilage erosion). The PBS group score increased gradually over time; the joint destruction gradually increased compared with the sham group. Studies on the improvement of joint structure in KOA mice by FJHQT decoction showed that FJHQT could significantly delay the early injury of KOA joint structure. The analysis of the articular cartilage area further proves that traditional Chinese medicine compound FJHQT decoction has a certain protective effect on KOA articular cartilage. It is well known that the main pathological change of KOA is the degeneration of articular cartilage. The pathological process of OA degeneration develops slowly, and gradually the articular cartilage is a connective tissue that is composed of a special extracellular matrix (ECM), which is mainly composed of aggrecan and type II collagen [24]. With the progress of OA disease, collagen type II is gradually reduced, and collagen fibers are gradually disordered. Inhibiting the degradation of type II collagen and aggrecan may play an active role in the treatment of KOA. We adopted immunohistochemical staining to detect the expression of collagen type II in the cartilage of knee joint [25]. Immunohistochemical analysis in this study showed that the FJHQ decoction group and Mobic group had a delay in the loss of type II collagen content in KOA mice for 4 and 6 weeks if compared with the PBS model group. Mobic (meloxicam) is an oral Cox-2 selective nonsteroidal anti-inflammatory drug that reduces persistent low levels of inflammation and is recommended by the international research association's guidelines for the treatment of osteoarthritis of the knee without other complications [4]. In chronic rat models of arthritis and spontaneous OA, Mobic has been shown to inhibit posterior paw swelling and bone and cartilage destruction [26, 27]. In this study, we used Mobic as a positive control drug. We found that compared with the PBS model group, the FJHQT decoction had a similar therapeutic effect as Mobic on early KOA. Compared with the PBS model group, the treatment of the Mobic group delayed the destruction of articular cartilage in KOA mice and improved the score of the joint structure. Our experimental results indicated that the FJHQT decoction has similar efficacy to Mobic in retarding KOA joint degeneration.

Lymphatic drainage is also closely related to the development of KOA. The development of KOA often involves the entire joint and does not only affect the cartilage. Increasing evidence has emphasized the relationship between the lymphatic system and the occurrence and development of KOA diseases [7, 8, 17, 18]. Improvement of lymphatic drainage is thought to be helpful in delaying the pathogenesis of osteoarthritis of the knee [8, 20, 21]. In this study, FJHQT decoction was used to improve the knee lymphatic drainage function of KOA mice to explore the correlation between lymphatic drainage function and KOA degeneration. Studies have reported that the function of lymphatic drainage plays an important role in joint diseases [28], and the dysfunction of lymphatic drainage in several animal models of arthritis indicates that the function of lymphatic drainage is negatively correlated with the severity of joint tissue injury [6, 13]. In order to detect the function of lymphatic drainage, we established the ICG-NIR lymphatic drainage imaging system in a previous study [11, 29, 30]. The indocyanine green-near-infrared (ICG-NIR) lymphatic imaging system was used to observe the drainage of lymphatic vessels around the knee joint [6, 31, 32]. The near-infrared light can penetrate deep into the tissue and visualize the fluorescence contrast agent in the tissue. The contrast between the light signal and the background fluorescence is very high [33]. The appropriate depth of penetration, good contrast, and spatial resolution, all provide the possibility to measure the characteristics of lymphatic contraction. However, due to the scattering of photons in the skin, NIR imaging is mainly used for macroanalysis [34]. The system provides a visual detection method for evaluation of the lymphatic drain function in animal experimental studies and for screening effective drugs to treat arthritis. Our study discovered that the FJHQT decoction can effectively promote early osteoarthritis knee ICG clearance in mice. Therefore, the FJHQT decoction is an effective drug to promote lymphatic drain function.

The lymphatic drainage system is one of the most important functions for removing the inflammation factor in KOA. Metabolic factors such as collagenase, metalloproteinase, cytokines, and chemokines are produced by various types of cells in the joints and released into the joints. These metabolic factors have adverse effects on cartilage and other tissue components [35] and are of great significance in cartilage degeneration [36]. Clearance of inflammatory cytokines is crucial to the alteration of KOA progression, and promoting lymphatic transport is a potential therapeutic target for improving KOA inflammation [13]. Metabolic factors are removed from the joints through lymphatic vessels, and adequate lymphatic drainage helps to improve inflammation, which is one of the key components of bone repair. The lymphatic reflux system plays a crucial role in this process. In this study, we found a correlation between lymphatic drainage function and mouse knee OARSI score, indicating that the occurrence and development of KOA are closely related to lymphatic drainage function. Therefore, we have reason to believe that the main function of FJHQT decoction is to promote lymphatic drain, unblock lymphatic vessels, and remove inflammatory factors, so as to reduce early KOA joint inflammation.

The ability of lymphatic drain is determined by a number of factors, including the clearance space of capillary lymphatic vessels for collecting fluid, mature lymphatic vessels, lymphatic metastasis within lymphatic vessels, lymphatic valves for controlling one-way flow, and lymph nodes for receiving lymphatic drainage [37–40]. Lymphatic drainage is affected by the permeability of the wall and the pumping capacity of the lymphatic vessels. Studies have shown that lymphatic vessels in inflammatory and invasive arthritis show increased frequency of lymphatic vessel pulsations in acute arthritis and return to normal in chronic arthritis [6]. The study also demonstrated the presence of two distinct lymphoid phenotypes in inflammatory arthritis: initially increased lymphoid transport and increased clearance, followed by reduced lymphoid pulsation and clearance in the chronic phase.

Studies have shown that inflammation is the body's defense response to pathogens or stimuli and is often associated with the formation of new blood vessels and lymphatic vessels. Inhibiting the formation of lymphatic vessels during the development of arthritis can aggravate the progression of arthritis [29]. Inflammatory stimulation of lymphangiogenesis is a compensatory mechanism that enhances clearance of inflammatory products [13]. Inflammation initially affects the products of lymphatic vessels, leading to various diseases [41]. In various disease models, the activation and increase of blood vessels are thought to increase the severity of inflammation [42–44], while lymphatic vessels play a beneficial role, possibly by promoting lymphatic clearance, reducing edema formation and levels of inflammatory mediators and the number of immune cells [39]. Inflammatory mediators in arthritis, such as prostaglandin IL-1*β*, IL-6, and TNF-alpha, directly affect the lymphatic function and reduce the frequency of lymphatic pumps [45, 46]. In addition, inflammatory mediators also affect lymphatic permeability, and in vitro evaluation of the effects of various inflammatory mediators on the monolayer of rat lymphatic endothelial cells, IL-6, TNF-alpha, and IFN-gamma significantly increased permeability [47]. Increased interstitial fluid pressure has been found to cause lymphangitic capillaries to dilate, thereby facilitating the entry of fluids and inflammatory cells into the lymphatic system, and thus clearing them from inflammatory tissues [39]. The complete signaling pathway involved in lymphangitic dilation and development consists of vascular endothelial growth factor receptor 3 (VEGFR-3) and its ligands VEGF-C and VEGF-D [48]. VEGF-C induces endothelial cell proliferation and migration, while VEGF-D can induce endothelial cell proliferation [39]. In addition to promoting or inhibiting lymphangitic dilation, lymphangitic clearance is also regulated by various signaling mediators, including TNF-alpha, IL-1*β*, histamine, and VEGFC/VEGFR-3 signaling pathways [49–51]. VEGF-C has an enhanced effect on lymphatic contraction and pumping ability, while various inflammatory mediators, including prostaglandin, histamine, and nitric oxide (NO), play a negative regulatory role [52–54].

During the KOA process, we also observed changes in the number of lymphatic vessels. In our previous study, we established a full-section scanning system to analyze the distribution and changes of lymphatic capillaries and mature lymphatic vessels in the knee sections [14]. Immunofluorescence staining can distinguish between capillary lymphatics and mature lymphatics. A large number of lymphatics exist in soft tissues around the knee joint [8, 14], mainly including synovial fat pad articular capsule ligament, patellar ligament, and other hard tissues such as meniscus cartilage and subchondral bone, and the number of mature lymphatics is significantly less than that of capillaries. Previous studies have observed an increase in the number of lymphatic joints in patients with chronic inflammatory arthritis or in mice [7, 18, 19]. A large number of new lymphatic vessels appear during the period of chronic arthritis [6], and inflammation can stimulate the increase of lymph nodes and lymphatic vessels in the surrounding tissues and regions [7]. With the development of OA, the number of lymphatic vessels gradually decreases, and the function of lymphatic drainage gradually declines [8]. These results indicate that OA has abnormal lymphatic structure and function. With the development of OA, the functional destruction of lymphatic vessels gradually worsens. In addition, a large number of clinical studies on the synovial membrane of the knee also prove that the synovial lymphatic density of OA patients decreases [17]. Increased lymphangiogenesis is involved in the compensatory response of joint inflammation, and inhibition of lymphangiogenesis can aggravate joint inflammation. In this study, it was found that FJHQT decoction promoted the increase of lymphatic vessels of the knee joint in early KOA mice. We also analyzed the correlation between the knee clearance rate OARSI score and the percentage of the capillary area and found a strong correlation between them. These results suggest that changes in the number of lymphatic vessels were also involved in the pathological process of KOA. However, we still need further studies to confirm whether FJHQT decoction can alleviate KOA joint inflammation by promoting lymphatic vessel production to delay KOA joint degeneration or not.

## 5. Conclusions

In summary, this study proved that FJHQT decoction could promote the generation of synovial lymphatic vessels in KOA joints, increase the function of lymphatic drainage, maintain the articular cartilage structure, and postpone the degeneration process of joints in the early stage of KOA, which may play a key role in the early-stage KOA's prevention and treatment.

## Data Availability

The data used to support the findings of this study are available from the corresponding author upon request.
